# Intracellular Transport of Silver and Gold Nanoparticles and Biological Responses: An Update

**DOI:** 10.3390/ijms19051305

**Published:** 2018-04-27

**Authors:** Elisa Panzarini, Stefania Mariano, Elisabetta Carata, Francesco Mura, Marco Rossi, Luciana Dini

**Affiliations:** 1 Department of Biological and Environmental Sciences and Technologies (DiSTeBA), University of Salento, 73100 Lecce, Italy; stefania.mariano@unisalento.it (S.M.); elisabetta.carata@unisalento.it (E.C.); luciana.dini@unisalento.it (L.D.); 2 Department of Basic and Applied Science to Engineering, Sapienza University of Rome, 00161 Rome, Italy; francesco.mura@uniroma1.it (F.M.); marco.rossi@uniroma1.it (M.R.); 3 Center for Nanotechnology Applied to Engineering of Sapienza (CNIS), Sapienza University of Rome, 00161 Rome, Italy; 4 CNR-Nanotec, Institute of Nanotechnology, via Monteroni, 73100 Lecce, Italy

**Keywords:** metal-nanoparticles, silver-nanoparticles, gold-nanoparticles, uptake, food, medicine, cellular responses, occupational exposure

## Abstract

Medicine, food, and cosmetics represent the new promising applications for silver (Ag) and gold (Au) nanoparticles (NPs). AgNPs are most commonly used in food and cosmetics; conversely, the main applications of gold NPs (AuNPs) are in the medical field. Thus, in view of the risk of accidentally or non-intended uptake of NPs deriving from the use of cosmetics, drugs, and food, the study of NPs–cell interactions represents a key question that puzzles researchers in both the nanomedicine and nanotoxicology fields. The response of cells starts when the NPs bind to the cell surface or when they are internalized. The amount and modality of their uptake depend on many and diverse parameters, such as NPs and cell types. Here, we discuss the state of the art of the knowledge and the uncertainties regarding the biological consequences of AgNPs and AuNPs, focusing on NPs cell uptake, location, and translocation. Finally, a section will be dedicated to the most currently available methods for qualitative and quantitative analysis of intracellular transport of metal NPs.

## 1. Introduction

Metal nanomaterials are universally considered as promising multifunctional platforms for wide purposes due to their peculiar photonic, electronic, catalytic, and therapeutic properties, versatile methods of synthesis ensuring wide size and shape features, and surface functionalization.

In the biomedical field, the most exploited metal-based NPs are silver (AgNPs) and gold (AuNPs) nanoparticles (NPs), the first due to their biocide activity, and the second for their photoactivation capability, inert character, biocompatibility, and easily and high yield of production [1,2]. They are efficiently used as therapeutic agents for cancer treatment and as medical tools for bioimaging and biosensing [3]. In the food sector, AuNPs are used as dietary supplements, whereas AgNPs are used in food packaging because of their antimicrobial properties. In particular, the antimicrobic activity of AgNPs is the reason for their increasing use in environmental treatments (e.g., air disinfection, water disinfection, groundwater and biological wastewater disinfection) and surface disinfection (e.g., silver-nanoparticle-embedded antimicrobial paints, antimicrobial surface functionalization of plastic catheters, antimicrobial gel formulation for topical use, antimicrobial packing paper for food preservation, silver-impregnated fabrics for clinical clothing) [4]. Consequently, the many consumer products in which AgNPs are present (estimated at 14% of products) are increasing the risk of exposure of humans—whose benefits and risks are reported by us in Panzarini 2017 [5]—and the AgNPs release into the environment, which in turn amplifies the possible interactions with animals, plants, and humans [6,7,8]. During the production and use of nanomaterials (NMs), very high is the possibility of exposure for workers, consumers and environment, but the derived effects cannot be precisely predicted because of the particulate and molecular identity of the nanoscaled materials [9]. Furthermore, there is the difficulty to identify companies producing or processing NMs, because many companies are not classified as nanotechnology companies [10].

A growing number of works directly or indirectly expose workers to probability to interact with NPs, and it is estimated that about 6 million workers will be potentially exposed to NPs in 2020, but there is still little data about the risks. The occupational activities with substantial probability of worker exposure to NPs identified by the European Agency for Safety and Health include construction, health care, energy, the automobile and aerospace industry, the chemical industry and electronics, and communication. It is very important to identify hazards from NMs impact and to define risks and strategies for preventing exposed workers. In general, humans become susceptible to NMs because of the lack of capability to tolerate and respond to these exogeneous toxicants relatively to inherited and genetic susceptibility, epigenetic induced modifications and age, pathological conditions, and lifestyle factors induced alterations, as recently reviewed by Iavicoli et al. [11]. Further, these factors are amplified by the great variability of NMs physicochemical properties that, in turn, dictate the response of humans to exposure. However, a systematic toxicity database regarding this does not still exist. The human body can come into contact with NMs of synthetic origin above all through three main ways: inhalation through the respiratory system, ingestion through the gastrointestinal tract, and absorption via the cutaneous route [12]. When NPs are able to overcome these barriers, further barriers protect internal organs in the human body. These internal or secondary barriers are the blood-brain barrier, which protects the brain, the blood-testicular barrier, which protects the male reproductive system, and the placenta, which protects the developing embryo.

Nanostructures, once inhaled, ingested, or administered topically, can reach the bloodstream and be transported and accumulated at the level of various organs. In vivo animal studies have shown that NPs can be located in the blood circulation and in the central nervous system (CNS), inducing inflammatory reactions at the pulmonary level and problems at the cardiovascular level [13] and beyond to accumulate in various organs such as liver, spleen, lymph nodes, and bone marrow [14].

Airways are one of the main routes by which the human body comes into contact, voluntarily or accidentally, with nanostructures. The deposition efficiency of the inhaled NPs depends mainly on their diameter and aerodynamic characteristics: in fact, size and shape are important for determining which compartment of the respiratory system will be mainly exposed between the upper airways, the lower airways or the alveoli. The particles, in general, are deposited efficiently in the entire respiratory tract, from the nasal cavity to the alveoli, through diffusional mechanisms [15]. The small NPs have the possibility to proceed more deeply in the respiratory system, to settle and be absorbed by the pulmonary epithelium entering the circulation, while those with a larger diameter are stopped at the upper respiratory cavity and expelled through mechanisms of mucociliary clearance [16].

Mucociliary transport is essential for clearance of the respiratory tract, while at the alveolar level, the NPs translocate via transcytosis through the epithelium of the respiratory tract. Here, they reach the pulmonary interstitium, where they can subsequently be phagocytized by alveolar macrophages or enter the bloodstream directly or via the lymphatic pathway [17].

Other studies suggest that inhaled NPs, after being deposited in the lungs, evade the control of alveolar macrophages and manage to infiltrate interstitial space, by translocation from the alveolar spaces through the epithelium [18]. Furthermore, the translocation of inhaled NPs to extra pulmonary sites, such as the circulatory system, the heart, the liver, and the brain [19], is possible, even if the mechanisms by which translocation occurs are not completely clarified.

The gastrointestinal route is potentially important for consumer, but it is considered less relevant for workers, at least in comparison to the pulmonary route. However, it is important to underline that a percentage of inhaled nanoparticles are cleared by mucociliar cells into the oral cavity and ingested into the gastrointestinal tract [20].

Also, skin contact with nanomaterials can also lead to adverse consequences. Estimates of possible dermal exposure to manufactured NMs in the workplaces have been reported. Certain metals, such as nickel, are also known to cause dermatitis. However, the three layers of skin (epidermis, dermis, and subcutaneous) make it difficult for ionic molecules to penetrate through. Furthermore, no evidences have been shown about the penetration through intact or damaged skin into systemic circulation [21]. 

Biomarkers, “chemicals, their metabolites, or the products of an interaction between a chemical and some molecules or cells that are measured in the human body” (Committee on Human Biomonitoring for Environmental Toxicants, National Research Council, 2006) have a great importance in occupational medicine, since they give information about the exposure over time and through different routes of exposure. Furthermore, they give advises about the toxicokinetics of several substances among workers.

When discussing on exposure assessment, there are three types of biomarkers that may be useful: exposure biomarkers, effect biomarkers and susceptibility biomarkers. Exposure biomarkers provide information on the route and on the source of exposure, such as assessment of a worker’s current exposure to solvents and some metals. Biomarkers of effect give an assessment of the consequence elicited by chemicals on physiological processes. They are indicators of an early health effect (possible health impairment; critical effect) or a clinical effect (disease). Susceptibility biomarkers indicate the relationship between the natural characteristics of an organism and the effects of exposure to a chemical. They can help to define the most critical moments when exposures can be more dangerous [22].

In general, biomarkers are usually measured in biological liquid, such as urine, saliva, and blood. For example, different studies have demonstrated the potential relevance of pulmonary cytochines as possible biomarker of effect for the evaluation of lung exposure to NMs [23]. However, this analysis is carried out by an invasive method, i.e., the sample of broncho-alveolar lavage liquid (BALF) and cannot be used as routine screening in humans. Recently, it has been shown that the presence of cytokines may be performed by a non-invasive procedure that analyzes the exhaled breath condensate [23].

Other data in literature report in experimental animals a comparison between the amount of metal NPs, such as silver and gold, and the concentration of the elemental metal found in the blood [9]. However, measuring the concentration of a metal in elemental form is not a correct way to evaluate a specific marker of exposure, because there would be an implication in the interpretation of data. Farther, the screening of relevant biomarkers of exposure is a difficult task for NMs compared to other substances, since there is not much information about their absorption, biodistribution and excretion.

The interactions of NPs with biological systems, including the entry into cells, play a key role in executing their functions and eventual toxicity. In fact, it is known that the NPs small size can allow an easy penetration into the cells and translocation among different cells, tissues, and organs that are remote from the portal of entry to the body, ultimately representing a great risk to human health.

Many routes are used by NPs to enter the human body, such as inhalation, ingestion, skin penetration and/or injection. At the cellular level, NPs can enter into cells through intracellular, i.e., phagocytosis, macropinocytosis, clathrin-mediated, caveolin-mediated, and non-clathrin and non-caveolin-mediated endocytosis, paracellular, and transcellular pathways [24].

The design of new biological functions or the prediction of the toxicological consequences of metal NPs in vivo first require the knowledge of their interplay, also accidental, with target cells and tissues that innately have barriers to prevent the entry of foreign particles. Physicochemical and mechanical characteristics of NPs, such as stability, size, surface charge, shape, hydrophobicity, surface chemistry, and protein and ligand conjugated, influence cellular internalization and trans-barrier transport. NPs–cell membrane interactions may also influence their intracellular trafficking, like sorting into different intracellular compartments, cellular retention, and biological fate, no matter if the final outcomes are adverse or favorable.

Thus, understanding the effects of various NPs characteristics on cellular and biological processes and manipulating the NPs characteristics could help in designing NPs efficient and safe, avoiding or facilitating internalization to better exploit the potentiality of the use of nanoconstructs. The presence of metal NPs in biological systems are a big concern and it remains difficult to give general warnings, since literature data are unlike in consequence of the NPs type, cell lines, experimental designs, and different endpoints of observations considered [25]. In addition, the lack of suitable user-friendly methodologies to investigate the extent and mode of NPs-cell interactions amplifies the scarcity of detailed investigations. Literature data concord that several hazardous effects occur at cellular level, like generation of reactive oxygen species, lipid peroxidation, genotoxicity and mutagenesis, apoptotic or necrotic cell deaths, mitochondrial dysfunction and changes in cell morphology [26].

Here, the existing knowledge and uncertainties regarding the biological consequences of the widely used Au and Ag NPs will be highlighted in relation to cellular uptake, localization and translocation of NPs. Moreover, a section will be dedicated to methods available for qualitative and quantitative analysis of cell-associated NPs, that allow to distinguish between cell surface bound and internalized NPs, and to follow NPs intracellular fate and speciation.

## 2. How Do Nanoparticles Fit in to the Cellular Uptake Process?

A significant issue to reduce possible dangers for human health or improve the efficacy of NPs, relies on the thorough knowledge of the biological interactions (cells, tissues, or organisms) and subsequent internalization [27]. Since cell membranes allow free diffusion only of small molecules (oxygen, carbon dioxide, water, and small hydrophobic or nonpolar molecules) or particles sized 10–30 nm, various distinct pathways for cellular internalization of particulate matter (lipids, proteins, glucose and other extracellular substances), pivotal for exerting an effect at a cellular level, exist. These pathways are categorized as endocytosis, a mechanism that internalizes cargo in transport vesicles derived from plasma membrane [28]. The endocytosis mechanisms include phagocytosis (uptake of particles by specialized cell types, i.e., macrophages, monocytes and neutrophils) and pinocytosis (uptake of extracellular fluids and soluble substances). Pinocytosis can be further divided into four mechanisms depending on the size of the vesicles and the protein involved in their formation. They include (1) macropinocytosis (an actin dependent pathway initiated with ruffling of the plasma membrane followed by large vacuoles named macropinosome); (2) clathrin-mediated endocytosis, also known as receptor-mediated endocytosis or RME (internalization of biomolecules via clathrin-coated vesicles containing plasma membrane specific receptors); (3) caveolae-mediated endocytosis (internalization of extracellular ligand and biomolecules through flask-shaped invaginations, named caveolae, consisting of the cholesterol-binding protein caveolin); (4) non-clathrin- and non-caveolin-mediated endocytosis [24].

In addition to the intracellular endocytic delivery system, vesicles, rather than undergoing degradation into cytoplasm, can be transported to the other end of cell surface and released into the extracellular environment. This route is known as transcellular delivery pathway. Finally, a passive process, named the paracellular delivery pathway, for transport of molecules is also known. The molecules transit between adjacent cells via tight junctions whose pores with diameters of up to 15 angstrom and negative charge regulate the delivery [29].

**Figure 1:**
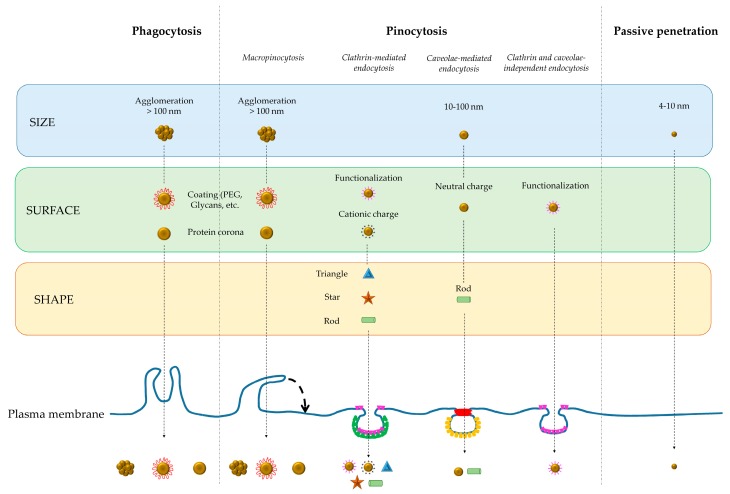
Internalization pathways of different types of nanoparticles. The cells can exploit different internalization mechanisms in relation to the nanoparticle properties, such as size, surface and shape. The cell can internalize the NPs by using different mechanisms also considering the same parameter.

Each type of NPs exhibits a preferred pathway for cellular internalization, and several investigations suggest that size, surface charge, shape, functionalization, and protein corona dictate entry and subsequent cytosolic access of NPs into living cells, as reported in Figure 1.

NPs size is the main feature affecting uptake, both pathway type and amount, that is strictly dependent on internalizing cells size, cell membrane tension and cell spreading. Also charge parameters of NPs are crucial for the NPs uptake: (a) cationic and neutral NPs are efficiently transported into the cells; (b) cationic NPs have a higher uptake than neutral ones; (c) neutral NPs are endocytosed in the caveolae-mediated pathway; (d) cationic NPs are transported using the paracellular pathway; (e) cationic NPs commonly use the clathrin-mediated pathway [30,31]. NPs shape strongly influences internalization and plays a key role in NPs designing [32]. Due to the curvature, spherical NPs have a higher internalization probability than asymmetrically shaped ones. In addition, the NPs shape dictates the accumulation in different organs or tissues. For example, in the lung discoidal-shaped NPs are internalized more than spherical or cylindrical ones; conversely, the liver cells internalize better the cylindrical NPs [33]. Finally, nano-worms are internalized by fibrosarcoma and breast cancer cells greater than spherical NPs [34]. Other important characteristics of NPs that can be modulated to positively or negatively affect uptake are surface properties. For example, it is worth noting that NPs used in medicine as drug delivery system require a great circulation time in the body for recognizing the specific sites of interest. Thus, the interaction of NPs with plasma proteins that can cause the opsonization of NPs leading to recruitment and clearance by immune cells stimulation [35] or the protein corona formation is very important. Modulation of hydrophobicity and hydrophilicity of NPs by adding molecules as polyethylene glycol (PEG) or zwitterionic agents allows to overcome this concern [36,37]. A strict correlation exists between uptake of metal NPs and cellular responses. In general, each type of NPs induces specific cell responses that are depending on the same parameters affecting the uptake, i.e., size, shape, surface functionalization and coating. In addition, NPs concentration, a parameter strictly dependent on the rate of uptake, plays a pivotal role for toxicity, that increases when high NPs concentration are used. Metal NPs preferentially enter into the cells via endocytosis. This route transports NPs from extracellular environment to endosomal and to lysosomal sites, thus changing dramatically the environmental conditions of the NPs. In fact, metal NPs pass from neutral pH (of about 7.4) of extracellular medium to nearly pH 6.0 of endosomes, to acidic pH (4.5) of lysosomes, which, in turn, trigger the release of relatively toxic ions in the cell [38]. Also, metal NPs once inside the cytoplasm can be degraded by cytoplasmic enzymes, like cathepsin L, that cause release of free metal ions and change in size and shape. The biodegradation affects cell homeostasis and functions, leading to damage of mitochondria, lysosomes, endoplasmic reticulum that, in turn, activate other mainstream adverse events leading to alteration of proteins, genotoxicity, reactive oxygen species (ROS) production, DNA damage, apoptotic and necrotic cell death, etc. [39]. For this reason, the mechanism of internalization is considered as a “Trojan horse effect” [40] and the release of toxic free ions by lysosomes as “lysosome-enhanced Trojan horse effect” [38]. The toxicity can be overcome by incubation with specific ions chelators, which do not affect the uptake efficiency and do not induce cross-toxicity [41], or by treatment with lysosomotropic agents neutralizing lysosomal acidity and, consequently, decreasing toxic ions release [42].

## 3. Gold Nanoparticles (AuNPs): Uptake Modality and Cellular Effects

As for all metal NPs, size, shape, and surface properties influence their cellular internalization [43]. Chan by using a set of AuNPs with size ranging between 10 to 100 nm, observed that 50 nm sized NPs show the highest uptake by human epitheloid cervix carcinoma (HeLa) cells demonstrating the dependence of uptake from size of NPs [44]. Similar results were obtained by Ko and coworkers that observed that 50 nm AuNPs are internalized by human adipose-derived stem cells more than 15, 30, 75, and 100 nm NPs [45]. Moreover, AuNPs may aggregate and the uptake efficiency of aggregates is controversial and dependent on cell type: in fact, the uptake rate is reduced in HeLa and A549 cells while it is increased in melanoma MDA-MB 435 cells respect to single and monodispersed NPs. The researchers explain this phenomenon because of different endocytosis pathways elicited by cells to engulf AuNPs: in particular, HeLa and A549 cells uptake AuNPs aggregates via receptor-mediated endocytosis, whereas MDA-MB435 via other receptor-independent mechanisms [46]. A few studies have shown enhanced cellular uptake of smaller 1–2 nm-gold nanoclusters (NCs) by Dendritic Cells (DCs) compared to a larger 12 nm gold NPs [47,48]. Conversely, Fytianos demonstrated DCs more efficiently internalize 50 nm gold NPs respect to 10 nm ones because of the minimum wrapping time required for 50 nm gold NPs compared to the smaller counterparts [49].

AuNPs coated with polyethylene glycol (PEG), polyvinyl alcohol (PVA) or a mixture of both (PEG+PVA)-AuNPs to have either positive or negative surface charge, display different behavior: monocyte-derived dendritic cells (MDDCs) internalize AuNPs but surface modification influenced the uptake amount. Limited uptake was observed for PEG-AuNPs, in contrast, (PEG+PVA)-AuNPs and PVA-AuNPs were largely internalized [50]. Moreover, by using several pharmacological inhibitors, Saha demonstrated that the uptake of cationic AuNPs in both cancer (HeLa) and normal (MCF10A) cells strongly depends on the AuNPs surface monolayer and involves different endocytic pathways as well as specific cell surface receptors (e.g., scavenger receptors) [51].

Most of the attention of researchers has been given to spherical AuNPs, but in recent decades different shapes of AuNPs have been synthetized to improve nanocarriers by modulating AuNPs chemical and physical properties depending on shape [52]. The new synthetized AuNPs include triangles [53], stars [54], cubes [55], octahedrons [56] plates and prisms [57]. In general, spherical AuNPs are efficiently taken up respect to other shapes. In fact, Cho [58] studied the effects of size, shape, and surface chemistry of spherical and cubic Au nanostructures (nanospheres and nanocages, respectively) whose surface is modified with poly(ethylene glycol) (PEG), antibody anti-HER2, or poly(allyamine hydrochloride) (PAA) on their uptake (including both adsorption and internalization) by SK-BR-3 breast cancer cells. The results show that both the size and the surface chemistry of the Au nanostructures influence their uptake by the cells: smaller AuNPs are better internalized than larger ones; PAA functionalized AuNPs are better internalized than anti-HER2 and PEG functionalized ones; cells internalize spherical particles over cubic particles when the surface was modified with PEG or anti-HER2 [58]. However, Nambara suggested that triangular AuNPs are more effectively taken up by RAW 264.7 macrophages and HeLa cells than spherical ones, even if with the same surface area and functionalization [59]. In June 2017, Xie and coworkers [60] synthetized three different shapes (stars, rods and triangles) of AuNPs to investigate the effects of shape on cellular uptake by RAW 264.7 macrophages. The Au nanoconstruct had the same size and the same coating (methylpolyethylene glycol-mPEG) to ensure the absence of other factors. In fact, mPEG allows a neutral surface charge, a well dispersion of AuNPs in aqueous solution and the prevention of adhesion of serum proteins. The highest cellular uptake by RAW 264.7 was observed with triangular shape after 24 h, followed by rods and stars. NPs are internalized as single particles and are localized in endosomes and/or lysosomes in the perinuclear region of the cells. However, different endocytosis pathways are engaged in relation to shapes: stars enter cells through clathrin-mediated process; rods are internalized through both clathrin- and caveolae-mediated endocytosis; triangles cause a strong cytoskeletal rearrangement leading the highest uptake and enter cells via clathrin-mediated endocytosis and dynamin-dependent pathway [60].

Despite the numerous studies about the influence of AuNPs parameters on cellular uptake, the role of cell size on uptake remains unclear. Wang investigated the influence of cell size on the cellular uptake of 50 nm sized PEG-AuNPs by using a method able to modify the size of human mesenchymal stem cells (hMSCs) through micropatterned PVA coated to obtain cells of 20, 40, 60 and 80 nm. Wang demonstrated that large-sized cells have a high total cellular uptake but a low average uptake/cells unit area, while small-sized hMSCs show opposite behaviors. In fact, the high total cellular uptake is due to the large contact area with the NPs; but the large size of cells causes a high membrane tension that requires a high wrapping energy for engulfing of NPs and thus reduces the average uptake/cells unit area [61].

Gold nanoparticles have been found to be very biocompatible and non-toxic according to many reports. Connor demonstrated that AuNPs of different sizes (4, 12, and 18 nm in diameter) and capping agents (citrate, glucose, biotin, etc.) enter into K562 human leukemia cells, do not induce any toxicity and reduce reactive oxygen species levels [62]. Similar results are achieved for other cell lines, such as Raw264.7 mouse macrophages [63] and dendritic cells [64]. In addition, beneficial cell responses exploiting in tissue engineering can be engaged by AuNPs. For example, modulation of the differentiation of stem cells by AuNPs leading to induction of differentiation and bones mineralization [65,66,67] and in immunotherapies and vaccine development by targeting DCs [68] has been demonstrated.

Even if AuNPs are considered as the highly compatible nanoconstructs, a potential toxicity mainly related to internalization modality has been demonstrated. A paper of Sabella et al. [38] demonstrated the release of free gold ions in monocytoid U937 cells, HeLa cells, human breast adenocarcinoma epithelial MCF7 cells, human colon adenocarcinoma epithelial Caco-2 cells, human neuroblastoma SHSy5Y cells and human hepatoma Huh-7 cells upon interaction with two different types of AuNPs. The two types of AuNPs have identical physico-chemical properties but differences in the ligand shell composition, a stripe like and a random distribution of the ligands named by authors as striped AuNPs and unstructured AuNPs respectively. Unstructured AuNPs enter cells via endocytic pathway and co-localize with lysosomes, while striped AuNPs are taken up via a non-endocytic pathway and mainly distribute in the cytosol. The authors demonstrated that the unstructured AuNPs are more toxic than striped ones suggesting that the induced NPs toxicity is internalization pathway-dependent. It is likely that unstructured AuNPs entrapped in the lysosomes undergo enhanced corrosion and ion leakage, with consequent toxicity to cells [38]. 

Goodman et al., demonstrated that gold nanoparticles are toxic against Cos-1 mammalian cells depending on surface charge: cationic NPs are toxic, conversely anionic ones do not be toxic [69]. Further, AuNPs are toxic when administered to endothelial SK-Mcl-28 and L929 cells [70] and HeLa cells [71].

## 4. Silver Nanoparticles (AgNPs): Uptake Modality and Cellular Effects

AgNPs easily pass the biologic barriers and can translocate from the route of exposure to other vital organs. The interaction between AgNPs and cells and uptake modality and biocompatibility, as already reported for AuNPs, is related to many factors related to both NPs, such as size, shape, surface charge, surface coating, solubility, concentration, and surface functionalization, and to experimental conditions or cells, e.g., distribution of particles, mode of entry, mode of action, growth media, exposure time, and cell type.

Primary brain astrocytes, normal human lung fibroblasts (IMR-90), and human glioblastoma cells (U251) internalize AgNPs through lysosomal or endosomal endocytosis [72,73]. Conversely, macrophages, fibroblasts, and glioblastoma cells uptake AgNPs via macropinocytosis, scavenger receptor and clathrin-mediated mechanisms [72,73,74]. Recently, Hsiao used three different brain cells (murine brain astrocyte-like ALT cells, murine microglial BV-2 cells and murine neuroblastoma N2a cells) to study the uptake and toxicity of 10 nm sized AgNPs. The uptake profiles are dose- and cell-dependent: ALT took up the highest amount of AgNPs, followed by BV-2 and N2a cells and cell viability correlates with the uptake levels. Moreover, lipopolysaccharide (LPS)-activated BV-2 cells took up larger amounts of AgNPs than their normal counterpart. Conversely no difference in NPs uptake between normal and LPS-activated ALT and N2a cells are detected. Caveolae-independent and clathrin-independent endocytosis, and phagocytosis are the preferred internalization pathways for ALT cells, while macropinocytosis and clathrin-dependent endocytosis are involved in uptake of BV-2 cells [75].

Depending on size and surface properties, once internalized, AgNPs translocate to the mitochondria and nucleus and elicit alteration of cell morphology, oxidative stress, DNA damage, inflammation, genotoxicity, mitochondrial dysfunction, and consequent induction of apoptosis or necrosis [76]. Smaller AgNPs exhibit an improved ability to pass the plasma membrane, localize inside the cell eliciting a higher toxicity, as demonstrated in spermatogonial stem cells [77]. In fact, uptake and cytotoxicity are amplified with smaller-sized AgNPs due to increased surface area and particle number for the same mass/volume dose, that in turn correlate with a higher rate of Ag^+^ ion release into the cell culture medium [78]. Conversely, in a recent study using AgNPs of 15, 50, and 100 nm, Chen et al. [79] demonstrated that 50 nm AgNPs exhibit the highest adsorption and passive uptake in red blood cells (RBCs), while the smallest 15 nm AgNPs are the most cytotoxic. Conversely, the 100 nm-sized AgNPs aggregate and are not able to pass the plasma membrane [79]. The high values in passage through the biological barriers of smaller sized AgNPs causes NPs accumulation that, in turn, elicits cytotoxicity in lung, stomach, breast and endothelial cells [78,80]. The shape of AgNPs plays a key role for the uptake and consequent cellular effect. Spherical AgNPs (30 nm) are efficiently endocytosed by A549 cells while very few silver wires (length: 1.5–25 μm; diameter 100–160 nm) are observed inside the cells. When cytotoxicity is considered, silver nanowires induce very high cytotoxicity compared to the minimal effects associated with silver nanospheres. This may be due to the interaction between silver nanowires and plasma membrane rather than the endocytosis mechanism [81]. To improve stability, dispensability, agglomeration, and impart novel functions to AgNPs, a panel of molecules can be used for coating the particles that in turn can also affect the cellular uptake. For example, uncoated AgNPs are largely uptaken by human lung cells respect to citrate/ polyvinylpyrrolidone (PVP)-coated ones because albumin and other human serum proteins suppress their cellular uptake. Conversely, the serum proteins enhance internalization of silica-coated NPs [78]. In general, this difference may be due to higher affinity of the negatively charged cell membranes respect to positively charged AgNPs, thus promoting internalization and intracellular bioavailability of these particles [82,83].

Zhang et al. [84] have recently reviewed cellular responses to AgNPs in in vitro models. There are several studies that dealt with the potential cytotoxicity and genotoxicity induced by AgNPs on both tumor cell lines and normal cell lines [78,85]. Cytotoxicity associated to AgNPs is related to oxidative stress and release of Ag^+^ ions deriving from dissolution of AgNPs [76]. Once Ag^+^ ions have been released, they interact with tiol groups of antioxidants, i.e., superoxide dismutase (SOD), thioredoxin and glutathione (GSH), causing oxidative stress, DNA damage up to apoptotic cell death [86].

Data in the literature report a correlation between mitochondrial damage and ROS production in cells [87]. AgNPs have a strong effect on mitochondria, leading to mitochondrial membrane potential drop, breaking of respiratory chain, oxidative stress and inhibition of ATP synthesis that give rise to the activation of apoptosis pathway [73,88]. Also, small AgNPs with a diameter of less than 10 nm are able to cross the nuclear pores, reaching the nucleus and leading to ROS production, DNA damage, cell cycle arrest, and chromosomal aberration in human fibroblasts and glioblastoma cells [73].

Furthermore, it has been observed that AgNPs induce genotoxic effects in HepG2 cells, human mesenchymal stem cells (hMSCs) and human peripheral blood mononuclear cells (PBMC). In particular, Kawata et al. [89], suggested that AgNPs cause dangerous effects on the DNA (e.g., chromosome aberration) by demonstrating the up-regulation of the DNA repair genes and the increase in micronuclei formation in cells treated with AGNPs low doses (<1.0 mg/L) [89,90].

AgNPs induce different effects in neurons leading to toxicity: alteration of cell morphology, degradation of cytoskeleton components, perturbations of pre- and postsynaptic proteins, and mitochondrial dysfunction [91]. Other studies indicate that AgNPs reduce cell viability in different cell lines by causing apoptosis through the mitochondrial pathway [92,93]; stimulate inflammatory and immunological responses in cells inducing cytotoxicity, elevated secretion of proinflammatory cytokines (such as interleukin-1β, inteleukin-2, tumor necrosis factor α, and prostaglandine E2) and increased blood-brain barrier permeability and immunotoxicity in a size-dependent manner in rat brain microvessel endothelial cells [94,95].

AgNPs have an important role also in angiogenesis. Gurunathan et al., showed that they act as anti-angiogenesis factor in cells by inducing the activation of phosphatidylinositol-3-kinase /Protein Kinase B (PI3K/Akt) pathway and inhibiting cell proliferation and migration mediated by vascular endothelial growth factor (VEGF) [92]. In another study, Sriram et al., demonstrated that AgNPs have an anti-cancer activity in Dalton’s lymphoma ascites cell line, in a dose-dependent manner AgNPs, by acting on caspase-3 activation and DNA fragmentation [96]. In addition, AgNPs inhibit HIF-1 expression and their downstream targets and this provides new evidences about the effects elicited by AgNPs on cytotoxicity mechanisms and angiogenesis.

Some dark sides remain about the effects of AgNPs on the development. Despite several studies demonstrated that various metal NPs have no significant effects on the morphology, viability and differentiation capability of stem cells, only few works reported the effect of AgNPs on human and non-human stem cells. In particular, it has been demonstrated that both AgNPs and Ag^+^ negatively impact the development by changing transcriptomic responses in embryonic stem cells (ESCs) due for AgNPs to the nanosized shape [97]. As already stated, the oxidative stress induction and free silver ions release mediate AgNPs cytotoxicity [98], even if it is not clear to which degree the toxicity depends on free Ag^+^ or AgNPs. To overcome ions release, AgNPs can be synthesized by design a specific surface capping and/or functionalization that, by interfering with dissolution process, limit or inhibit the release of Ag^+^. Recently, among surface coatings, such as starch, PVP (poly(*N*-vinyl-2-pirrolidone), citrate, polymers, etc. there is an increasing interest in using carbohydrates as biomimetic molecules because of their double function: (i) glycans allow synthesis of NPs without toxic chemicals traces; (ii) NPs glycans capping serves as targeting molecules and mediates cellular responses [99]. The advantages in using glucose, fructose or sucrose to form a capping around 30 nm AgNPs that affect their internalization have been reported by us in different cell types (lymphocytes, HeLa and HepG2 cells) [100,101,102,103,104,105]. In particular, glucose has been demonstrated a key factor in inducing uptake of AgNPs [106].

Endosomes and lysosomes are the main organelles target of AgNPs [76]. AgNPs, by interacting with the acidic lysosomal compartment, induce ROS production, including superoxide anions (O^2−^), hydroxyl radicals (^•^OH), and hydrogen peroxide (H_2_O_2_), production. Thus, ROS diffusion into the cytoplasm results in oxidative damage to proteins and other organelles, such as mitochondria. In particular, H_2_O_2_ dissolutes AgNPs and causes accumulation of Ag^+^ in lysosomes. AgNPs and Ag^+^ can escape from lysosomes, amplifying the increase of ROS in cytoplasm, that, in turn, allow further dissolution of AgNPs with Ag^+^ production. ROS can also mediate the release of Ca^2+^ from endoplasmic reticulum (ER) leading to imbalance of calcium homeostasis [73]. In this manner four death pathways are elicited. The first one is the necrotic pathway via rupture of the plasma membrane; the second is the induction of mitochondrion-dependent apoptosis, via alteration of electron transfer; the rupture of lysosomal membrane is the cause of the third death pathway, the lysosome-mediated apoptosis; the last one is the ER-mediated apoptosis. Moreover, AgNPs present in cytoplasm can diffuse into the nucleus through nuclear pores and directly damage DNA and chromosomes, while free Ag^+^ ions released by the AgNPs that have entered the nucleus can contribute to damage DNA [73].

## 5. Methods for Qualitative and Quantitative Analysis of Cell-Associated Nanoparticles

The study of nanoparticle–cell interactions is a key question in the fields of nanomedicine as well as in nanotoxicology. In fact, the amount of nanoparticles internalized by cells or bound to the external surfaces of cells determines the NPs toxic profile while cellular binding and uptake of medically effective NPs decide their efficiency and efficacy. Despite their importance, these processes are under investigated, mainly due to the lack of suitable user-friendly methodologies. The researchers concord that an ideal methodology would require minimal sample preparation, allow sufficient resolution to assess NPs cellular and subcellular localization at the single cell level, allow high sample throughput, and finally be independent of material properties not requiring fluorescent or radioactive labeling of NPs.

Several methods, reviewed in Ivask et al. [107], can be employed to study the cellular uptake, distribution and speciation of metal NPs in cells. Here, we highlight only the most useful techniques for a direct observation of metal NPs, and for the quantitative evaluation of biodistribution, focusing on our experience in the detection of AgNPs and AuNPs.

The most common method to visualize silver and gold NPs at a high magnification is electron microscopy, namely transmission (TEM) and scanning (SEM). Even if TEM analysis is a time-consuming technique, its advantage is that it does not require specifically tagged metal NPs, as for example it is needed for fluorescence microscopy, since metallic materials possess higher electron density and can easily visualized under electron beam. Moreover, to increasing the contrast of imaging, dark field observation can be used [108]. Dark-field microscopy has been widely used to visualize interactions between mammalian cells and AgNPs in in vitro but also in in vivo experiments [109]. Anderson et al. [110] used enhanced dark field microscopy to visualize NPs in tissues of animals that were exposed to 20 nm AgNPs and Roth et al. [111] used the same methodology to identify NPs in animals pre-exposed to nanoparticulate metal oxide.

Electron microscopy can be used also for the quantification of metal NPs, even if this is really a time-consuming method. In fact, Mass Spectrometry-Based (MS) methods, such as inductively coupled plasma mass spectroscopy (ICP-MS), atomic emission, and optical emission mass spectrometry (AES and OES) are becoming increasingly popular in quantifying cell-associated metal NPs [112]. Although these methods are very sensitive with detection limits in the order of parts-per-billion, MS methods are not able to detect any changes in speciation that may take place during biological exposure but enable only elemental analysis. Time-resolved or single-particle ICP-MS (SP-ICP-MS) encompasses the limitation of ICP. Today, SP-ICP-MS is of growing popularity for sensitive (detection limit in ng/L range) characterization of metal NPs [113,114] in the field of environmental chemistry as well as in nanotoxicology. SP-ICP-MS has been used to analyze AgNPs and AuNPs in organisms [115], tissues [116] and to study the dynamics of AgNPs transformations in human plasma [117]. Finally, Raman microspectroscopy imaging represents a powerful bioanalytical method to provide information regarding the chemical composition of a single cell without prior staining. This technique has been used to visualize and map both AgNPs [118] and AuNPs [119] inside the cells.

To distinguish between cell surface bound and internalized NPs is pivotal in studying the relationship cells-NPs. The most common techniques employed for this purpose are confocal fluorescence microscopy, TEM and SEM. In particular, TEM allows to reconstruct 3D images and determine the NPs cellular localization by sequential ultrathin specimen sections [120]. Field emission (FE) SEM can also be used to distinguish between intracellular and externally bound NPs with a resolution of 1 nm [121], by using different accelerator voltages [122]. In addition, electron microscopic techniques can be coupled with energy dispersive X-ray (EDX) analysis, and backscattered electron imaging (FE-SEM) to offer an ideal analytical platform for the characterization of NPs in and on the cells [123,124]. Using FE-SEM, NPs images can be acquired inside the cells without disruption of the cellular shape, and also the initial steps of NPs incorporation into the cells can be captured. In addition, the matrix used to treat the glass renders the sample highly stable under the required accelerating voltage. By coupling EDX analysis, an analytical technique used for the elemental analysis or chemical characterization based on the interaction of X-ray with the sample, it is possible to confirm the presence of nanoparticles inside the cells.

Havrdova [125] first reported the potentiality of FE-SEM for imaging superparamagnetic iron oxide nanoparticles inside hMSCs. FE-SEM allows us to better observe NPs inside the cells but avoids the complex procedure including contrast staining and metal coating. Further, the researchers demonstrated that nanoparticles inside cells can be mapped using FE-SEM as the nanoparticles protrude through the membrane during the dehydration and drying sample preparation steps [125].

By using dark and bright field TEM, coupled with EDX analysis and ICP-MS analysis we studied the uptake and cellular distribution of in vivo AgNPs and AuNPs solutions injected in the mouse tail vein during experiments performed to evaluate the localization of NPs in designing a nanoconstruct to be used in cancer therapies.

**Figure 2:**
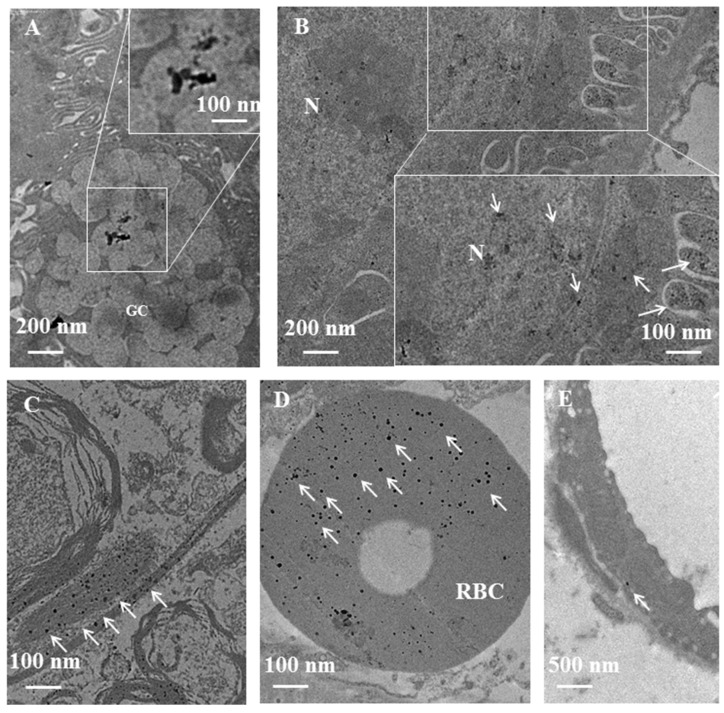
Biodistribution of AgNP-G in a murine model after injection of 10^8^ NPs into tail vein. Transmission electron microscopy micrographs of ultrathin sections of mouse organs. White arrows indicate AgNPs as single NPs or as cluster. N: nucleus; GC: goblet cell; RBC: red blood cell; (**A**) intestine; (**B**) kidney; (**C**) brain; (**D**) red blood cell in kidney; (**E**) endothelial cell.

**Figure 3:**
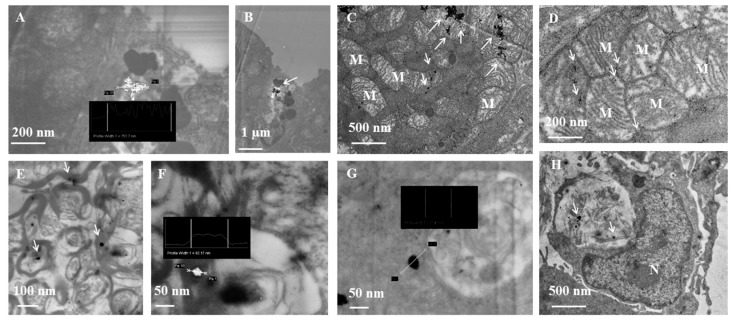
Biodistribution of AuNPs in a murine model after injection of 108 NPs in tail vein. Scanning transmission electron microscopy (STEM) micrographs of ultrathin sections of mouse organs. White arrows indicate AgNPs as single NPs or as cluster. N: nucleus; M: mitochondria. (**A**,**B**) liver (dark field); (**C**,**D**) heart; (**E**,**F**) brain (dark field (**E**), bright field (**F**)); (**G**) kidney (bright field); (**H**) macrophage in the connective tissue in intestinal villi.

Table 1 reports the NPs biodistribution and the semiquantitative analysis of the amount of NPs retained by the various mouse organs. In general, AgNPs and AuNPs were accumulated in larger quantities inside the mouse organs (Table 1). However, the organs internalizing NPs, and the NPs amounts differ between AgNPs or AuNPs. Indeed, AuNPs are widely distributed in terms of organs and amount than AgNPs. This is probably due the small size of NPs, 10 vs. 30 nm, that permits to pass biological barrier, including blood brain barrier. The kidney and the brain are the organs where we detected the more quantity of AuNPs. We observed AgNPs also in red blood cells of the kidney and in endothelial cells. Probably, these data show that gold nanoparticles moved to the kidney, as expected, since the kidney physiological function is filtering the entire blood flow through the fenestrated endothelium of glomerular capillaries. Different is the localization of AgNPs and AuNPs in the liver: AgNPs are internalized by hepatocytes, while AuNPs by Kupffer and endothelial cells. It is well known that the sinusoidal capillaries in the liver are fenestrated (50–180 nm) and lined with the Kupffer cells, which rapidly uptake AuNPs; conversely, we have hypothesized that AgNPs are probably trapped in the Disse space and can be taken up by hepatocytes. The mechanism is still not well understood. Low amount of AuNPs was detected in the pancreas, while AgNPs number is low in the brain and in the spleen. The analysis of intracellular localization shows that NPs taken up by the cells are found mainly inside mitochondria, nucleus, rough endoplasmic reticulum (RER) as single nanoparticles or smaller clusters. Dark and bright field analysis confirms the presence of NPs inside the cells. In Figure 2 and Figure 3 we report TEM micrographs of mouse organs sections after NPs injection.

In addition to the extent of cellular interaction and (intra)cellular localization, various transformations that may take place after the entry of NPs into the cells are important for evaluation of the NPs cellular effects. Only synchrotron methods are truly capable of such analysis. Synchrotron X-ray absorption spectroscopy (XAS) is one of the few methods capable for elemental speciation analysis since it allows to obtain information regarding the oxidation state, symmetry and identity of the coordinating ligand environment for an element of interest by exploiting the ability to tune the energy of incident X-rays. This technique does not require any pretreatment of samples or extraction/isolation of the NPs as can be conducted in situ or in vivo on hydrated cells by using cryo conditions to reduce the risks of artifacts caused by the intensity of the X-ray beam. Gräfe [126] has published a review describing the potential of synchrotron XAS methods in metal speciation analysis in 2014.

## 6. Conclusions

The study of metallic materials is one of the most ancient scientific fields, as their properties—including strength, toughness, thermal and electrical conductivities, ductility, and high melting point—make metals useful for a wide range of applications, mainly based on the bulk metallic properties. New applications exploit the novel fascinating metal nanomaterials properties, which are size-, shape-, and crystal form–dependent. Metal nanomaterials have a long history of preparation and applications, the field has undergone explosive growth only in recent years as reviewed in Gentile et al. [127]. Metal-based nanostructured materials are used in a variety of food- and medical-related applications. For example, silver-based nano-engineered materials are currently the most common nanocomposites used in food packaging for their antimicrobial capacity, while Au-based NPs are the most common used nanoconstructs in medicine, as drug carrier and imaging tools, due to their chemical/physical properties and biocompatibility. A strong correlation exists between uptake of nanomaterials and biological responses. Our analysis indicates that changes in the size, shape and surface features will affect their cellular uptake, including modality and amount and intracellular fate, that, in turn, elicit a positive or a negative response by cells. Thus, it is possible to envisage several strategies to obtain safer metal NPs: (a) design of specific coatings able to skip or ignite the endocytosis; (b) design of specific procedures to escape or reach the organelles as lysosomes; (c) design a surface coating resistant to acidic pH of lysosomes to avoid free metal ions release. The first two strategies along with functionalization of NPs for specific targeting into the cell cytosol are particularly important in medicine. Conversely, the third strategy represents an approach of industrial interest to realize biocompatible metal NPs useful in food sector. This will lead to a better interactions of human body whit nanomaterials and ease the safety of metal-based NPs also in consideration that release of nano-engineered materials may occur in the workplace and uncertainties still exist regarding several aspects of the risk posed by NPs for workers. In fact, the very few data available suggest that more severe adverse health effects than those caused by larger particles or bulk material may be expected.

## Figures and Tables

**Table 1:** Semiquantitative analysis of the biodistribution, as amount and localization, of AgNPs and AuNPs after injection in mouse tail vein of 10^8^ NPs performed by transmission electron microscope.

	AgNPs (30 nm, d-Glucose Coated, Round)		AuNPs (10 nm, PEG+FDG Coated, Round)	
Organs	Amount	Localization	Amount	Localization
Liver	++	Nucleus and cytoplasm of hepatocytes	++	Kupffer cells and endothelial cells (nucleus)
Kidney	++	Cortex level	+++	Red blood cells and cortex
Intestine	++	Goblet’s cells mucus Cytosol of enterocytes	++	Goblet’s cells Connective tissue
Brain	+	Schwann cells: myelin	+++	Schwann cells: myelin
Spleen	+	Nucleus, mithocondria, RER	++	Endothelium of blood vessels Nucleus, mitochondria, RER
Stomach	++	Cytoplasm of stomach cells	N.D.	
Heart	++	Mithocondria, lysosomes, cytoplasm	++	Mitochondria, lysosomes, cytoplasm
Pancreas	N.D.		+	RER

(+) low amount of NPs; (++) high amount of NPs; (+++) highest amount of NPs; N.D.: not detected.
